# Effects of formalin fixation on polarimetric properties of brain tissue: fresh or fixed?

**DOI:** 10.1117/1.NPh.10.2.025009

**Published:** 2023-05-24

**Authors:** Romain Gros, Omar Rodríguez-Núñez, Leonard Felger, Stefano Moriconi, Richard McKinley, Angelo Pierangelo, Tatiana Novikova, Erik Vassella, Philippe Schucht, Ekkehard Hewer, Theoni Maragkou

**Affiliations:** a University of Bern, Institute of Tissue Medicine and Pathology, Bern, Switzerland; b University of Bern, Graduate School for Cellular and Biomedical Sciences, Bern, Switzerland; c Bern University Hospital, University of Bern, Department of Neurosurgery, Inselspital, Bern, Switzerland; d University of Bern, Inselspital, Bern University Hospital, University Institute of Diagnostic and Interventional Radiology, Support Center for Advanced Neuroimaging, Bern, Switzerland; e IP Paris, École Polytechnique, CNRS, LPICM, Palaiseau, France; f Lausanne University Hospital and University of Lausanne, Institute of Pathology, Lausanne, Switzerland

**Keywords:** formalin fixation, brain tissue, Mueller polarimetry, image segmentation, neuropathology, neurosurgery

## Abstract

**Significance:**

Imaging Mueller polarimetry (IMP) appears as a promising technique for real-time delineation of healthy and neoplastic tissue during neurosurgery. The training of machine learning algorithms used for the image post-processing requires large data sets typically derived from the measurements of formalin-fixed brain sections. However, the success of the transfer of such algorithms from fixed to fresh brain tissue depends on the degree of alterations of polarimetric properties induced by formalin fixation (FF).

**Aim:**

Comprehensive studies were performed on the FF induced changes in fresh pig brain tissue polarimetric properties.

**Approach:**

Polarimetric properties of pig brain were assessed in 30 coronal thick sections before and after FF using a wide-field IMP system. The width of the uncertainty region between gray and white matter was also estimated.

**Results:**

The depolarization increased by 5% in gray matter and remained constant in white matter following FF, whereas the linear retardance decreased by 27% in gray matter and by 28% in white matter after FF. The visual contrast between gray and white matter and fiber tracking remained preserved after FF. Tissue shrinkage induced by FF did not have a significant effect on the uncertainty region width.

**Conclusions:**

Similar polarimetric properties were observed in both fresh and fixed brain tissues, indicating a high potential for transfer learning.

## 1 Introduction

Gliomas form a heterogeneous group of tumors of the central nervous system (CNS), and most of them are characterized by a diffuse infiltrative growth of cells in the preexistent parenchyma of the CNS.^1^^–^^3^ The optimal treatment approach is radical tumor resection while preserving neurological function, followed by radio- and chemotherapy for malignant brain tumors.^4^^–^^6^ For this purpose, it is of great importance to accurately detect the borders between healthy and neoplastic brain tissue. In addition there is a survival benefit for removing the infiltration zone around the solid tumor.^7^ However, these areas may contain fibers essential for specific neurological function, such as speech, vision, sensibility, and cognition. Knowing the orientation of fibers in-sight during surgery would contribute to saving essential fibers while sacrificing non-essential ones. Current intraoperative state-of-the-art imaging modalities appear to present limitations and fail to provide this crucial information.^8^^–^^11^

Polarimetric imaging presents great potential as a fast and non-contact diagnostic tool for tissue characterization. Several studies already proved polarimetry to be beneficial as a non-invasive technique for evaluating dermatologic diseases,^12^^,^^13^ the risk of pre-term labor,^14^^,^^15^ the presence of Aβ amyloid plaques observed in Alzheimer’s disease,^16^ and for differentiating healthy from neoplastic tissue zones in various human tissues, such as colon, skin, and cervix, both *ex vivo*^17^^–^^20^ and *in vivo*.^21^^–^^23^

In a previous study, we showed that wide-field imaging Mueller polarimetry (IMP) presents many advantages that can be harnessed to improve brain tumor delineation.^24^ Linear birefringence is observed in nerve fibers and is created by the highly ordered structures found in the white matter and forming fiber tracts, the bundles of axons, as well as by the myelin sheath surrounding the nerve fibers.^25^^,^^26^ Interestingly, our prior studies demonstrated that assessment of linear birefringence through IMP can be used to detect in-plane nerve fiber orientation and to differentiate white matter, containing these nerve fibers, from gray matter in fresh calf and fixed human thick brain sections^24^ using machine learning algorithms.^27^ The implemented wide-field IMP system was also proved to be robust in surgery-like settings,^28^ highlighting an important translational potential for this technique.

Once enough brain imaging data have been collected, machine learning (ML) models can prove to be helpful for the automatic and accurate segmentation of brain images.^29^ Such an approach is typically required to perform high-quality image annotations in a fast manner. This is particularly important for an *in vivo* delineation tool, to fulfill the necessity of obtaining fastly accurate segmentation. Moreover, ML algorithms can improve the diagnostic value of the imaging modalities and speed up the analyses, for example, by helping in the detection of neurodegenerative diseases,^30^^–^^33^ by allowing the detection and classification of multiple demyelinating diseases^34^^,^^35^ or by enabling fast and accurate classification of tumors^36^^–^^40^ from magnetic resonance imaging (MRI) data. Polarization imaged based ML frameworks were developed to accurately classify different tissue types^41^ and neoplastic lesions in colon,^42^ cervix,^43^ and skin.^44^ Numerous tissue samples are required for acquiring the training data sets for the image post-processing ML algorithms. Fresh human brain tissue is notoriously difficult to obtain and it must be handled correctly in an experimental setting. Research experiments are therefore often performed on formalin-fixed brain specimens. Tissue fixation provides many advantages, such as arrest of the decay process, easier handling, good preservation of the morphological structures and bioactive moieties,^45^ and more importantly is compatible with downstream histological applications.^33^^,^^46^^,^^47^ The most commonly used fixative agent is formalin (10% formaldehyde), a cross-linking agent creating bonds between soluble proteins. Cross-linkage is a relatively fast process; it is completed by 24 to 48 h after propagation of formalin in the tissue.^48^ Tissues appear slightly different under white light before and after formalin fixation (FF); therefore, a more comprehensive analysis of the polarimetric properties changes induced by FF appears essential to feed the ML segmentation algorithms with tissue-specific polarimetric properties. This would ideally facilitate the optimal delineation with automatic machine learning ML paradigms leveraging transfer learning concepts, which refers to the process of using pre-trained models pre-trained on one task to improve the performance on another related task with limited data. A possibility involves training algorithms using the easily accessible FF tissue measurements, and then transfer these algorithms to fresh tissue data. Transfer learning is known to be feasible when matching populations exhibit slight variations in the observed samples,^49^^,^^50^ which could be the case for the variations of polarimetric parameters between fresh and fixed tissue. However, it appears important to understand to what extent the standard FF method impacts the polarimetric properties of brain tissue and, hence, to assess the feasibility of transfer learning applications. This has been previously assessed for pig myocardium and liver^51^ as well as for human oral cancerous tissue^52^ but in our knowledge not for brain tissue. The FF is also reported to cause physiological shrinkage in tissues from a number of organs.^53^^–^^58^ During diagnostics such changes are of significant importance, especially when reporting tumor size. Furthermore, it would be absolutely relevant to determine the extent of shrinkage in brain tissue, as well as to explore the potential consequences on the developed algorithms.

In this study, we evaluated the effects of FF on the depolarization, linear retardance, and azimuth of the optical axis of fresh pig brain tissue using the multi-spectral wide-field IMP system operating in reflection. More precisely, we (a) quantified the differences in the parameters of depolarization, linear retardance, and azimuth of the optical axis induced by FF, and (b) evaluated the tissue shrinkage caused by the fixation process. The results were analyzed with a focus on the possible implications for further image segmentation algorithm development, and more specifically on the potential transfer learning of ML algorithms from fixed to fresh domain.

## 2 Methods

### 2.1 Imaging Mueller Polarimetry System

We studied fresh and fixed animal brain thick sections using the custom-built multi-spectral wide-field imaging Mueller polarimetric system operating in reflection configuration in the visible wavelength range (450 to 700 nm). The IMP systems that acquire the complete Mueller matrix (MM) of a sample have been successfully used for the characterization and medical diagnosis of different pathologies.^14^^,^^16^^,^^18^^–^^21^ In this study, all presented measurements were performed at 550 nm. The details on the design, calibration, and optimization of our IMP instrument have been already reported elsewhere.^20^^,^^28^^,^^59^^–^^61^ For the sake of completeness, we remind the basic principles of our IMP system operation. The incident light beam produced by a Xenon (SOPRO Comeg 230) source passes through the polarization state generator (PSG) before interacting with a sample. The PSG consists in an assembly of fixed polarizer and two bistable V-shaped ferroelectric liquid crystals (FLCs) in smectic C phase^62^ operating as waveplates with fixed linear retardance and variable azimuth that can be electrically controlled. A further waveplate is placed between the two FLCs.^59^ After reflection/scattering by a sample, the light beam passes through the polarization state analyzer (PSA), which is made from the same optical components as PSG but assembled in a reverse order, before reaching the camera (Stingray F080B ASG). The modulation of polarization of probing light beam is performed sequentially. To reconstruct the MM of a sample, 16 intensity measurements have to be performed (4 linearly independent polarization states generated by the PSG that are analysed with the 4 linearly independent polarization states of PSA). To increase the signal to noise ratio, the series of 16 intensity measurements has been performed and averaged 8 times. The measurement accuracy was guaranteed by implementing the eigenvalue calibration procedure.^63^

The post-processing of the recorded MM images was done with the Lu-Chipman polar decomposition algorithm^64^ that was applied pixel-wise. The calculated maps of the depolarization, linear retardance, and azimuth of the optical axis of the brain specimens were obtained afterwards, as described in Rodriguez-Nunez et al.^28^ The azimuth of the optical axis was used to assess the effects on the orientation of the fiber tracts before and after the FF of the same specimen.^24^ The depolarization and linear retardance have been proved to be informative for *ex vivo* brain tissue differentiation,^24^^,^^27^ and hence, we decided to focus our quantitative work on the examination of the images of these three parameters.

### 2.2 Brain Samples and Measurement Protocol

**Fig. 1:**
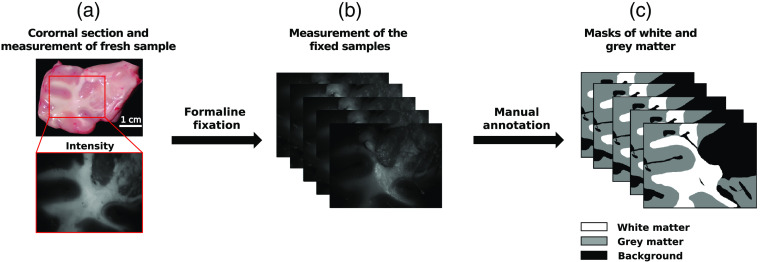
The pipeline of the measurement and annotation process. (a) Color photo of a 3-cm-thick pig coronal section slice (top) and gray-scale reflected intensity image of the central zone shown by a red rectangle (bottom), (b) measurements were performed on the same section after FF (see text), and (c) gray and white matter masks were created manually from gray-scale reflected intensity images.

Fresh cadaveric pig brain hemispheres were obtained from a local butcher shop 6 hrs post-mortem. A total of 10 brain hemispheres were used for this study to ensure the study of images from a diverse set of brains. The brains were dissected at the level of corpus callosum and brainstem in the midline. Thirty coronal sections, each ∼3  cm thick, were obtained using a scalpel [Fig. 1(a)] and placed flat in a glass Petri dish. For the measurements, sections were selected based on whether the quality of the tissue was sufficient. The quality requirements included (i) absence of tissue deterioration and cutting artifacts and (ii) presence of both gray and white matter in the imaged zone of a specimen. The coronal sections were obtained from multiple brain areas (some sections were occipital while others were frontal) to achieve a global representation of brain tissue in the samples.

We used the wide field IMP system and Lu-Chipman decomposition of measured MM images to produce the maps of the depolarization, of the linear retardance, and of the azimuth of the optical axis of the thick coronal sections of fresh brain tissue. This first measurement was performed on fresh brain tissue right before the fixation of the sections and represents the first time point (0 h). The sections were fixed by being placed in small plastic recipients containing formalin (10% formaldehyde). For the measurements performed after FF, the samples were removed from formalin, washed with distilled water, and then measured. For accurately assessing the time evolution of the polarimetric parameters after FF, four sections were measured at five different time points following FF: +12 hrs, +24 hrs, +36 hrs, +48 hrs, +7 days, making up a total of 6 time points for which the parameters were obtained. A visual qualitative comparison of the depolarization, of the linear retardance, and of the azimuth of the optical axis maps was performed for all measurements of the same sample at different time points. Thirty fresh sections and 30 fixed sections measured with the polarimeter 10 days post-fixation [Fig. 1(b)] were selected for the assessment of the polarimetric values in the border between gray and white matter (described in Sec. 2.4). Finally, both gray and white matter masks were annotated manually for all measurements [Fig. 1(c)].

### 2.3 Quantitative Analysis

**Fig. 2:**
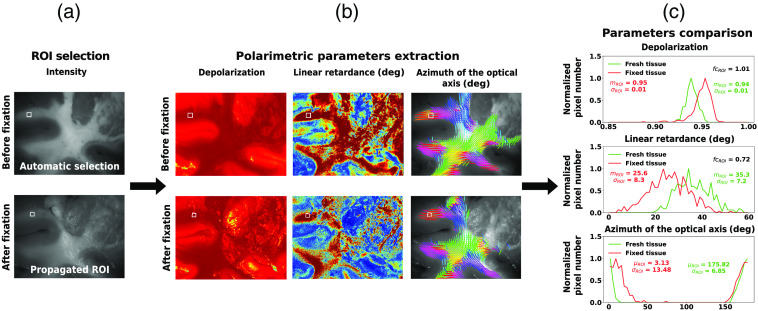
The sequence of main steps in the quantitative analysis of the fixation effects. (a) ROIs (white square box is shown as a typical example) were selected automatically (see text) in the gray-scale reflected intensity image of fresh tissue using the masks generated in Fig. 1. These masks were then propagated to the images of FF tissue using a co-registration pipeline on both gray-scale reflected intensity images. (b) The polarimetric parameters of depolarization, linear retardance, and azimuth of the optical axis were then extracted using the ROIs generated in the previous steps. (c) Statistical data describing the distributions of polarimetric parameters for both fresh and FF tissues were then obtained and compared.

To quantitatively analyze the evolution of polarimetric properties before and after fixation, we generated automatically and randomly, to avoid any bias linked to a manual selection, 25 squared regions of interest (ROIs) (by selecting with an uniform random variable a point in the image that we used as top left corner of the ROI), each 20×20  pixels, completely within gray matter, and 25 ROIs, each 20×20  pixel, within white matter, using the corresponding fresh measurement tissue gray-scale reflected intensity image [Fig. 2(a)]. The use of small ROIs to analyze the local effect of FF is necessary because of the spatial inhomogeneity of the polarimetric parameters in the tissue. The size of the square was chosen to be 20×20  pixels to analyze the effects of FF for local brain tissue regions while keeping the number of pixels (400) large enough for robust statistical analyses. The gray-scale reflected intensity images at the different fixation time points were co-registered with the fresh one, using a custom pipeline based on Elastix^65^^,^^66^ to ensure the analysis of the same areas in the images acquired after FF. The gray-scale reflected intensity image of fresh tissue was registered to the images of FF tissue [Fig. 2(a)], allowing to propagate the selected ROIs on the images of FF tissue. The ROIs registered as gray/white matter in the images of fresh tissue and as white/gray matter or background [defined as neither white nor gray matter, Fig. 1(c)] in the images of FF tissue were removed for further analysis. The histograms of the depolarization, linear retardance, and azimuth of the optical axis were then extracted for each ROI [Fig. 2(b)]. For the first two parameters, the medians $m_{\mathrm{ROI}}$ were computed for each ROI [Fig. 2(c)] and used as a descriptive statistics, based on the presence of skewed distributions within single ROIs (Fig. S1 in the Supplementary Material). The value of $m_{\mathrm{ROI}}$ for the fixed tissue was then divided by the corresponding value for the fresh tissue, to obtain the fold change $fc_{\mathrm{ROI}}$ in the medians. The mean and standard deviation of the $fc_{\mathrm{ROI}}$ values across the images were then obtained for both depolarization and linear retardance and compared. The angular nature of the azimuth of the optical axis prevented using a similar comparison. We computed the difference $\theta_{\mathrm{ROI}}$ in the mean angle observed before and after fixation in each ROI as a metrics. The mean and standard deviation of the $\theta_{\mathrm{ROI}}$ values across the images were then obtained and compared. Statistical differences between the means of the fold changes and the angle differences at the different fixation time points were assessed using SciPy’s Mann-Whitney U test (v1.9.3).^67^ For depolarization and linear retardance, the medians $m_{\mathrm{ROI}}$ were also averaged across the four images obtained at each time point to check for the preservation of the general contrast between gray and white matter after FF. All the error bars represented in the result plots correspond to the 95% confidence intervals.

### 2.4 Segmentation Analysis

**Fig. 3:**
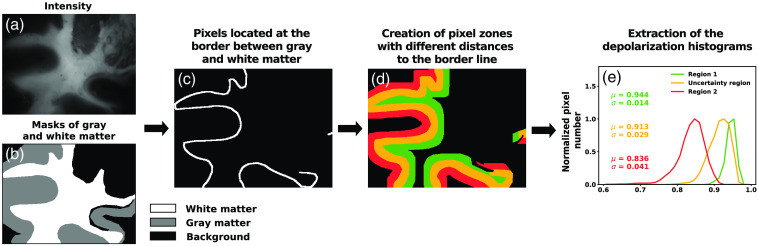
The illustration of the main steps used to investigate the behaviour of the depolarization values at the border region. (a) Gray-scale reflected intensity image of a pig coronal section for which (b) masks of gray and white matter were manually drawn (see Sec. 2.2). (c) The pixels located at the border region between gray and white matter were selected. (d) These pixels were then dilated to create regions of fixed size at a different distance of the border region (here only three regions are represented for the sake of simplicity). (e) The polarimetric parameters were then extracted for the different regions, and descriptive statistical values were further computed and compared.

For analyzing the behavior of the tissue at the gray/white matter border region, we reused the previously created masks of gray and white matter [Figs. 3(a) and 3(b)] and generated a line of the pixels located at the border by checking pixels assigned to the white matter located next to pixels assigned to gray matter [Fig. 3(c)]. We subsequently dilated this line using OpenCV (v 4.6.0.66)^68^ with a number of six iterations and the kernel, being a 3×3 identity matrix, for a total of nine times [Fig. 3(d), here only three regions were represented for the sake of simplicity]. Sequential regions, consisting of the pixels incorporated during the current dilation step and absent from the previous one, were generated through this process. These regions were therefore located further and further away from the border region. The pixels included in the newly dilated regions were considered for the analysis in case they belonged to either white or gray matter labeled regions but were discarded in case they belonged to the background. The depolarization values were then extracted for these regions and combined across different images. Descriptive statistical values, such as the mean μ and the standard deviation σ, were computed and compared between the regions defined previously [Fig. 3(e)]. The position of each region was then correlated to a physical distance related to the border region using the following equivalence relations

$$\text{Field of view}\;(\mathrm{FoV})=516\times 388\;\text{pixels}\approx 24\times 20\;{\mathrm{mm}}^{2}.$$ (1)

Thus, the size of a pixel in the x and y axis corresponds to

$$x_{\text{pixel size}}\approx \frac{24}{516}\approx 0.047\;\mathrm{mm},$$ (2)

$$y_{\text{pixel size}}\approx \frac{20}{388}\approx 0.051\;\mathrm{mm}.$$ (3)

We then aimed to determine the so-called uncertainty region, which would correspond to the region in which the parameters are not similar to the white matter ones nor the gray matter ones (see Sec. 3.3). For that purpose, the means μ in the regions aforementioned were fitted with a sigmoid using SciPy’s curve fit function (v1.9.3).^67^ We then used the kneed package (v0.8.1)^69^ to define the location of the two elbows on the curve corresponding to the transition point between white matter and uncertainty region and between uncertainty region and gray matter, on the left and right sides of the plots [see Figs. 7(c) and 7(d)], respectively. The estimated width of the uncertainty region, defined as the difference between the two elbow points, was then computed.

## 3 Results

### 3.1 General Effects of Formalin Fixation on Polarimetric Properties of Brain Tissue

**Fig. 4:**
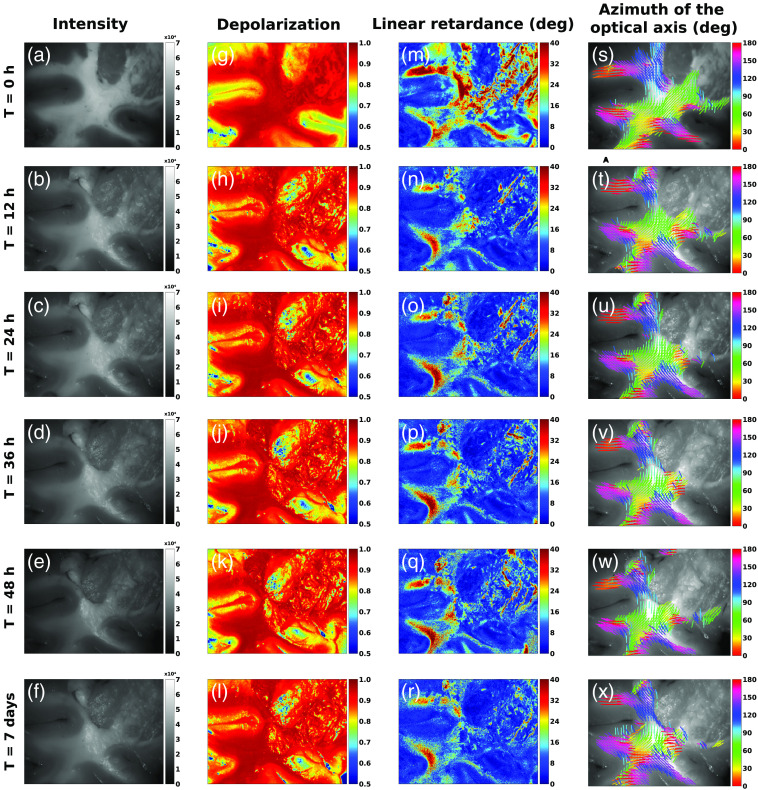
Polarimetric maps calculated from the experimental MM images of coronal sections of pig brain at different fixation time points (0 hrs, +12 hrs, +24 hrs, +36 hrs, +48 hrs, and +7 days). Each row represents the polarimetric maps acquired for a time point. The gray-scale reflect intensity (a)–(f) images and the polarimetric maps of depolarization (g-l), linear retardance (m)–(r), and azimuth of the optical axis (s)–(x) are demonstrated. The colored lines depicting the orientation of the optical axis were plotted in the regions, where all three conditions were fulfilled: the region was assigned as gray or white matter (see Sec. 2.2 for more details), the values of linear retardance >4  deg, depolarization >90% and gray-scale reflected intensity > mean intensity.

The polarimetric maps of depolarization, linear retardance, and azimuth of the optical axis obtained during the measurements of coronal sections of a pig brain at different times post-fixation are shown in Fig. 4. The gray-scale reflected intensity images [Figs. 4(a)–4(f)] were used to generate the masks of gray and white matter (see Sec. 2.2), as from the gray-scale reflected intensity image and with basic knowledge of neuroanatomy it is possible to differentiate between two types of brain tissue. One can also appreciate the apparition of physiological tissue shrinkage and tissue deformation, especially in the white matter region following FF. It should also be noted that specular reflections appear on the right-hand side of the image following FF, possibly as a consequence of the tissue deformation. In the depolarization maps [Figs. 4(g)–4(l)], we observed an important difference in the value of this parameter between gray and white matter. The former presents a lower depolarization than the latter, allowing contrast visualization of both tissue types. The same pattern of contrast remained visible after FF for all different time points at which the measurements were performed. Moreover, it was possible to observe the contrast between both tissue types using the linear retardance maps [Figs. 4(m)–4(r)], with the white matter displaying higher values than the gray matter. However, the contrast between gray and white matter in the retardance maps was substantially reduced by the fixation process compared to the depolarization maps. Finally, the azimuth of the optical axis maps was used to track nerve fibers in white matter of brain tissue, as described in previous studies.^24^ Slight changes in the maps of the azimuth were detected after FF. Most of the observed changed is believed be due to tissue shrinkage and deformation (described in Sec. 3.3), especially after 7 days. The FF did not disturb significantly the azimuth values in the examined images of brain sections [Figs. 4(s)–4(x)] preserving the orientation of the fiber tracts. Consequently, it seems that even if minor changes are induced by FF, especially in linear retardance values, they do not greatly impair the contrast between the two tissue types and do not perturb fiber orientation identification.

### 3.2 Quantitative Analysis of Polarimetric Parameters After Formalin Fixation

**Fig. 5:**
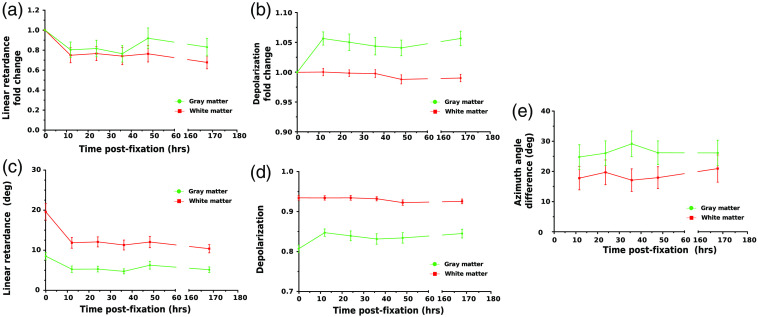
Evolution of the values of polarimetric parameters with time after FF. The mean value and 95% confidence intervals of the fold changes computed for areas of 20×20  pixels are plotted with respect to post-fixation time for (a) linear retardance and (b) depolarization. The mean value and 95% confidence intervals of the local medians for areas of 20×20  pixels are plotted with respect to post-fixation time for (c) linear retardance and (d) depolarization. (e) Plot of the evolution of the average of the difference in the value of the azimuth of the optical axis in 20×20  pixels areas before and after fixation with respect to fixation time.

The next step consisted of quantifying the differences in the polarimetric parameters induced by FF. To do so, we selected 25 areas (20×20  pixels each) within the gray and white matter zones in the gray-scale reflected intensity images of four coronal sections of pig brain. The fold changes in the median of the parameters were then obtained (see Sec. 2.3). Their evolution with time is represented in Fig. 5(a) for the linear retardance and Fig. 5(b) for the depolarization. There was an important evolution in the linear retardance fold change, when comparing fresh and 12 hrs-fixed tissue, for both gray (U = 1600, $n_{1}=85$, $n_{2}=100$, $p_{\text{value}}=2\times 10^{-15}$) and white matter (U = 1400, $n_{1}=94$, $n_{2}=100$, $p_{\text{value}}=1\times 10^{-19}$). The magnitude of the change was a reduction of about ∼27% in gray matter and ∼28% in white matter. Interestingly, little to no change of values was observed in linear retardance after the first two measurements (Table 1). For the depolarization parameter, there was a statistically significant change of fold change, when comparing fresh and fixed tissue, for gray (U = 7800, $n_{1}=85$, $n_{2}=100$, $p_{\text{value}}=1\times 10^{-26}$) but not for white matter (U = 4900, $n_{1}=94$, $n_{2}=100$, $p_{\text{value}}=0.78$). The magnitude of the change was rather small, an increase in about ∼5% for the gray matter and virtually no change for the white matter. Furthermore, there were small to no changes for the subsequent measurements after the initial one (Table 2). Although two value changes were found to be statistically significant for the depolarization in the white matter zone (+48 hrs, U = 5417, $n_{1}=94$, $n_{2}=96$, $p_{\text{value}}=6\times 10^{-3}$ and (+ 7 days, U = 5332, $n_{1}=94$, $n_{2}=96$, $p_{\text{value}}=0.035$), the magnitude of the observed change was minimal with a maximum of 1% of depolarization value. The averages of the medians in each ROI were also obtained to verify the stability of the contrast between gray and white matter (see Sec. 2.3). Their evolutions are represented in Fig. 5(c) for the linear retardance and Fig. 5(d) for the depolarization. Despite a change in the polarimetric parameters, the contrast between gray and white matter was still present after fixation. Similar conclusions to the ones obtained with the fold change analysis could be drawn when comparing the averages of the medians (Table S1 and S2 in the Supplementary Material). Thus, both the depolarization and the linear retardance seem to be useful polarimetric markers to differentiate gray from white matter in both fresh and fixed brain tissues. The average of the differences in the mean azimuth of the optical axis angle observed before and after fixation for each ROI is represented in Fig. 5(e). There was an important change in the observed azimuth angle, when comparing fresh and 12 hrs-fixed tissue, for both gray (U = 8500, $n_{1}=85$, $n_{2}=100$, $p_{\text{value}}=3\times 10^{-37}$) and white matter (U = 9400, $n_{1}=94$, $n_{2}=100$, $p_{\text{value}}=3\times 10^{-38}$). However, there was no statistically significant variation in the mean azimuth angle difference observed for later time points (Table 3). The observed variation was expected and could be explained by the fact that the sample was placed and aligned manually at each measurement time point with the position mismatch of a few degrees. This is also indicated by the important values of standard deviations reported. The tissue morphological changes induced by FF also explain the observed changes. The mean azimuth angle difference was larger for gray matter, which is expected, because the absence of highly organized brain fibers in gray matter results in low retardance values, thus, impacting the orientation calculations. Globally, these results indicate that the time of fixation did not amplify the observed changes when comparing fresh and fixed tissue and that the contrast between gray and white matter observed in the images of fresh brain tissue is still preserved in the images of brain tissue after FF.

**Table 1:** Summary of the statistics for the comparison between the linear retardance fold change mean values at different time points post-fixation. All the reported U and p values correspond to the test of the null hypothesis, stating that the mean variable is the same as the one 12 hrs post-fixation.

Time	Mean fold change	U	$n_{1}$	$n_{2}$	$p_{\mathrm{val}}$
**Linear retardance fold change in gray matter**					
**0 hrs**	1	1600	85	100	$2\times 10^{-15}$
**+12 hrs**	0.73±0.43	3612	85	85	1
**+24 hrs**	0.76±0.46	3613	85	87	0.80
**+36 hrs**	0.71±0.45	3899	85	86	0.45
**+48 hrs**	0.87±0.58	3453	85	92	0.18
**+7 days**	0.76±0.48	3722	85	89	0.86
**Linear retardance fold change in white matter**					
**0 hrs**	1	1400	94	100	$1\times 10^{-19}$
**+12 hrs**	0.72±0.42	4418	94	94	1
**+24 hrs**	0.74±0.41	4117	94	91	0.66
**+36 hrs**	0.72±0.48	4494	94	92	0.64
**+48 hrs**	0.73±0.48	4490	94	96	0.95
**+7 days**	0.64±0.39	5113	94	96	0.11

**Table 2:** Summary of the statistics for the comparison between the depolarization fold change mean values at different time points post-fixation. All the reported U and p values correspond to the test of the null hypothesis, stating that the mean variable is the same as the one 12 hrs post-fixation.

Time	Mean fold change	U	$n_{1}$	$n_{2}$	$p_{\mathrm{val}}$
**Depolarization fold change in gray matter**					
**0 hrs**	1	7800	85	100	$1\times 10^{-26}$
**+12 hrs**	1.056±0.048	3612	85	85	1
**+24 hrs**	1.050±0.062	3862	85	87	0.61
**+36 hrs**	1.041±0.066	4061	85	86	0.21
**+48 hrs**	1.040±0.061	4439	85	92	0.12
**+7 days**	1.056±0.055	3780	85	89	0.99
**Depolarization fold change in white matter**					
**0 hrs**	1	4900	94	100	0.78
**+12 hrs**	1.000±0.029	4418	94	94	1
**+24 hrs**	0.998±0.025	4196	94	91	0.82
**+36 hrs**	0.998±0.031	4611	94	92	0.44
**+48 hrs**	0.987±0.036	5417	94	96	$6\times 10^{-3}$
**+7 days**	0.991±0.030	5332	94	96	0.035

**Table 3:** Summary of the statistics for the comparison between the azimuth angle differences at different time points post-fixation. All the reported U and p values correspond to the test of the null hypothesis, stating that the mean variable is the same as the one 12 hrs post-fixation.

Time	Mean angle difference	U	$n_{1}$	$n_{2}$	$p_{\text{val}}$
**Azimuth of the optical axis in gray matter**					
**0 hrs**	0	8500	85	100	$3\times 10^{-37}$
**+12 hrs**	24.3±19.1	3612	85	85	1
**+24 hrs**	25.5±19.0	3512	85	87	0.57
**+36 hrs**	28.7±19.9	3144	85	86	0.11
**+48 hrs**	26.0±18.9	3675	85	92	0.49
**+7 days**	26.1±19.5	3556	85	89	0.50
**Azimuth of the optical axis in white matter**					
**0 hrs**	1	9400	94	100	$3\times 10^{-38}$
**+12 hrs**	17.6±18.6	4418	94	94	1
**+24 hrs**	19.5±19.1	3914	94	91	0.32
**+36 hrs**	17.5±18.1	4281	94	92	0.91
**+48 hrs**	17.7±18.0	4333	94	96	0.64
**+7 days**	20.7±22.1	4183	94	96	0.39

### 3.3 Manual Segmentation of Gray and White Matter

**Fig. 6:**
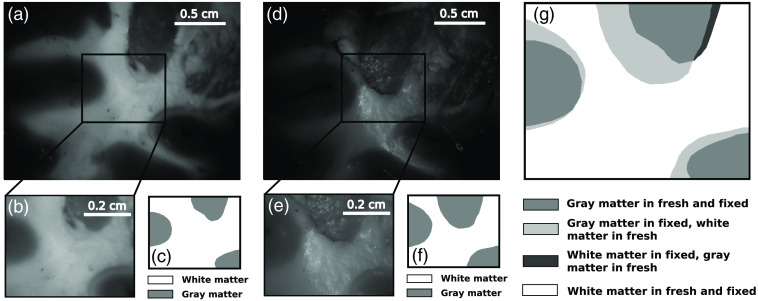
Illustration of the of tissue shrinkage following FF. (a) Gray-scale reflected intensity image of the fresh coronal brain section and (b) zoom on a specific sub-region (160×126  pixels) of the gray-scale reflected intensity image and (c) corresponding labels. (d) Gray-scale reflected intensity image of the same region after FF (t=+48  hrs) and (e) zoom on a specific sub-region (160×126  pixels) of the gray-scale reflected intensity image and (f) corresponding labels; (g) two overlapped binary masks for gray and white matter that were created from the binary masks (c) and (f).

The gray-scale reflected intensity images [Figs. 4(a)–4(f)] suggested the presence of tissue shrinkage and deformation induced by the FF process. This is mostly visible in Figs. 6(a)–6(f), where brain tissue considerably shrinks following FF. This was indicated by the presence of pixels that were attributed manually to white matter zone before FF and to gray matter zone after FF [see, for example, the pixels rendered in white, Fig. 6(g)]. They represent 13.9% of the total amount of pixels in the image.

**Fig. 7:**
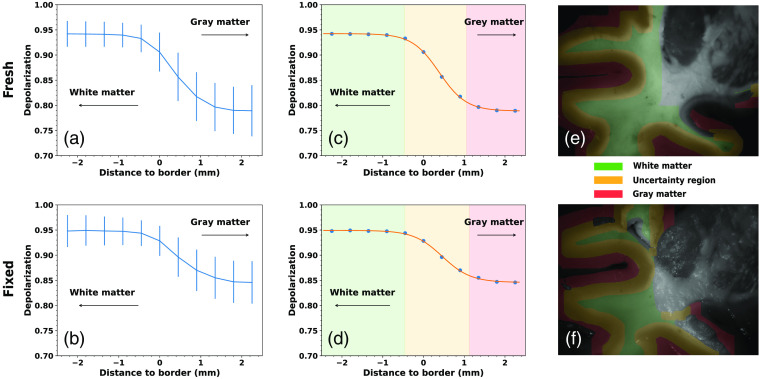
Determination of the width uncertainty region for both fresh and fixed brain tissue using data of polarimetric measurements. Mean and standard deviation of the depolarization values for the regions described in Sec. 2.4, plotted against distance to the physical gray and white matter border for fresh (a) and fixed (b) tissue (*n* = 30 for both fresh and fixed brain tissue measurements). Estimation of the uncertainty region was done using a fitted sigmoid curve for the depolarization data for fresh (c) and fixed (d) tissue. Over-imposition of the gray matter, uncertainty region and white matter on sample gray-scale reflected images for fresh (e) and fixed (f) tissue.

To investigate the effect of such a shrinkage on polarimetric parameters, the depolarization values averaged over the dilated border zones (see details in Sec. 2.4) are plotted against the distance to the center of the dilated region from the initial border line for the fresh and fixed brain tissue measurements in Figs. 7(a) and 7(b), respectively. There appears to be an uncertainty region where it is not clear whether we are detecting gray or white matter, making the segmentation ground truth somewhat unclear in this area. As the data points obtained were able to be represented by a “S”-shaped curve, with the value of depolarization in gray and white matter being the two plateau regions, we fitted a sigmoid function the depolarization data for both fresh and fixed brain tissue [Figs. 7(c) and 7(d), respectively]. The best-fit sigmoid functions had the following coefficients:

$${\text{sigmoid}}_{\text{fresh}}(x)=\frac{-0.15}{1+\exp(-3.02\times (x-0.39))}+0.94,$$ (4)

$${\text{sigmoid}}_{\text{fixed}}(x)=\frac{-0.10}{1+\exp(-2.87\times (x-0.46))}+0.95.$$ (5)

We then aimed to determine the width of the uncertainty region, which was estimated to be 1.53 mm for fresh tissue and 1.59 mm for fixed tissue. This finding suggested that despite the tissue’s physiological shrinkage after FF, the size of the so-called uncertainty region remains almost constant. The uncertainty region is represented over-imposed on the gray-scale reflected images for both fresh tissue [Fig. 7(e)] and fixed tissue [Fig. 7(f)]. In summary, we report the existence of an uncertainty region of a width close to 1.55 mm, where the depolarization values are changing continuously when moving from gray to white matter. We also prove that the size of this uncertainty region does not differ between fresh and fixed brain tissues.

## 4 Discussion

In our study, we report that the depolarization maps calculated with the Lu-Chipman decomposition of the complete MM images provide the visual contrast between the gray and white matter zones in both fresh and fixed brain tissue images. This contrast is even more pronounced in the depolarization maps of fresh brain tissue. The same observation applies for the maps of the linear retardance, the second parameter of choice revealing a contrast between gray and white matter in both fresh and fixed brain tissues. The drop in linear retardance values induced by FF was estimated to be 27% in the gray matter and 28% in the white matter, whereas the depolarization value increased by 5% in the gray matter and remained constant within white matter. The values of the azimuth of the optical axis, an indicator of the orientation of white matter fiber tracts, were affected by FF. The mean deviation in the angles observed was found to be 25 deg in gray matter and 18 deg in white matter. However, despite these variations, fiber tracking still appeared possible for white matter, indicating that this polarimetric parameter remains a reliable optical marker for brain fiber tracking in both fresh and fixed brain tissues. Interestingly, there was no change in the values of both parameters, depolarization, and linear retardance, after 12 hrs of fixation, indicating that FF time did not amplify any of the observed changes. Delineation of two brain tissue types with a small spatial error margin is relevant for numerous applications *in vivo*, such as the accurate differentiation of tumor tissue during neurosurgery.^24^ We showed that despite the physiological tissue shrinkage observed in thick coronal sections of brain after FF, the size of this spatial error margin was not significantly modified by FF. This finding is important for the anticipated translation of the algorithms of segmentation of gray and white matter developed and trained on FF brain polarimetric data to *in vivo* applications.

Wood et al.^51^ have reported an increase in depolarization values by 25% and 50% after FF for pig myocardium and liver, respectively. In our experiments, we report an increase in the depolarization values by 5% in the gray matter zone and no change in the white matter zone of pig brain tissue after FF. For the linear retardance values, it was found to increase by ∼10% and ∼30% in pig myocardium and liver, respectively.^51^ Conversely, we found a decrease in the scalar retardance values after FF of 27% for gray and 28% in white matter of pig brain. As highlighted by Wood et al.,^51^ these results suggest that the impact of FF on polarimetric properties is highly tissue dependent and should be opportunely analyzed for the specific tissue types. Several reasons might explain the difference in the observed results. First, it should be noted that the system used by Wood et al.^51^ works in transmission, whereas our system operates in reflection and uses a different wavelength (635 versus 550 nm). Second, in some cases, the optical anisotropy observed in biological tissues can be explained by the presence and composition of extracellular matrix (ECM).^70^ Collagen fibers are present in the ECM of a variety of organs, including liver and myocardium, whereas in the brain they mostly are found in the meninges and vasculature.^71^ The brain’s ECM is a macromolecular network composed of proteins and polysaccharides betweens neurons and glial cells, and the optical anisotropy observed in the brain is due to the myelin sheath surrounding the nerve fibers.^25^^,^^26^ This different organ composition most probably contributes to the distinct polarimetric parameter evolution observed between these organs. Finally, Wood et al.^51^ mentioned that the observed increase in both linear retardance and depolarization could partially be explained by the small volume increase measured after FF, resulting in a longer optical path length of the detected signal. On the contrary, in our study, we noted a tissue shrinkage after FF. Different trends for the polarimetric parameters with FF in our study and that of Wood et al.^51^ are possibly due to a combination of all these three factors.

Despite the presence of changes in the polarimetric parameter values following FF, the contrast between different brain tissue types observed in fresh tissue is preserved in fixed tissue as well. High polarimetric contrast between the different types of brain tissue is desired for the segmentation tasks, including gray and white matter delineation in the polarimetric images of brain measured in reflection geometry images. McKinley et al.^27^ reported on the generation of the similar delineation between gray and white matter zones of fixed brain tissue by leveraging both the depolarization and the linear retardance maps, in conjunction with the gray-scale reflected intensity images. Transfer learning is feasible when matching populations exhibit slight variations in the observed samples. Recent studies made use of transfer learning for medical images segmentation.^49^^,^^50^^,^^72^ It was also shown that transfer learning was useful in the context of segmentation and classification of MRI data acquired using different scanners, especially in the presence of slight alterations and (non-)linear intensity deviations in the obtained images.^73^^–^^76^ Overall, these studies suggest that slight variations in the data distribution might be corrected with various learning methods and strategies. A comprehensive analysis was required to determine the effect of FF on the polarimetric properties distributions, to estimate the feasibility of transfer learning approaches in this context, and to also supply the segmentation algorithms with tissue-specific polarimetric properties. Considering the estimated polarimetric parameter changes with FF for brain tissue, transfer learning relating fixed and fresh tissue polarimetric data would constitute a viable option also for polarimetric image segmentation and classification purposes and might only require minor fine tuning for the intensities values and for the polarimetric parameters values in fresh brain tissue. However, potential limitations to the straightforward application of transfer learning from fresh to fixed brain tissue remain. In particular, spatial deformation caused by the tissue shrinkage after FF were observed and reported for neoplastic tissue in various organs.^53^^–^^58^^,^^77^^,^^78^ Formalin diffuses through the tissue and binds to protein amino groups, creating extensive cross-linked proteins and nucleic acids.^79^ While this process prevents biological tissues from decay, it might however introduce histological changes, such as cell shrinkage or distortion, which might in turn cause shrinkage to the whole specimen. Such an effect has been reported in the kidney,^53^^,^^54^ skin,^55^ prostate,^56^^,^^57^ and observed in the optic nerve.^58^ The magnitudes of the reported shrinkages were different in each case. Interestingly, no shrinkage was reported in neoplastic breast tissue after fixation.^80^ The extent of shrinkage was also estimated in the human brainstem^77^ and in human complete brains,^78^ with a reported decrease in longitudinal distances of 1% to 8% for the former and a volume shrinkage of 48% and length shrinkage of 20% for the latter, respectively. Our qualitative observations were in line with the literature findings, with a clearly visible shrinkage on both white and gray matter regions of our specimens.

The decrease in linear retardance values after FF combined with almost constant depolarization values that are not affected by FF, might also be explained by the volumetric shrinkage of brain tissue after FF. Indeed, the drop of the linear retardance values is mainly observed in the central zone of the specimen [see Figs. 4(m)–4(r)], containing two stacks of crossing fibers, with a vertically oriented fiber tract (depicted in blue and green from top to bottom) overimposed on two horizontally oriented fiber tracts (mostly depicted in red and purple). Following FF, volumetric shrinkage of brain tissue will reduce the thickness of the overlying layer of fibers, which are oriented differently with respect to the underlying layer fibers. We believe that this phenomenon might be responsible for the diminished linear retardance values, as probing light beam may reach the underlying crossing fibers and partially compensate for the phase shift accumulated when passing through the top layer of fibers. The depolarization values were not modified as they do not depend on the orientation of the fibers in a stack.

The size of the uncertainty region, corresponding to the region in space where the depolarization values are in between the typical values for gray and white matter, was not affected by FF in our experiments. With this view, the estimation of such uncertainty region would therefore represent a relevant spatial conditioning prior for the tissue segmentation model underlying the polarimetric-specific transfer learning. Such valuable information could be also injected in fine-tuned registration algorithms to optimally recover spatial deformations introduced by FF.

## 5 Conclusion

Our findings demonstrate stability of polarimetric parameters (depolarization, linear retardance, and azimuth of the optical axis) after FF for both gray and white matter of brain tissue. It supports the assumption that formalin-fixed brain specimens are good surrogates for scarcely available fresh human tissue when testing IMP systems. This is a significant step toward routine laboratory use of formalin fixed tumor samples, which are more accessible than fresh samples. Large-scale tests and measurements on fixed samples followed by re-checking on few fresh samples will accelerate the design and training of tumor segmentation algorithms. The impact of other histological procedures (such as freezing of sections or paraffin-embedding and shrinkage of brain tissue) on polarimetric parameters of brain tissue should also be investigated in the future allowing a more comprehensive understanding.

## Data, Materials, and Code Availability

All the codes used for data analysis and data generated in this study have been deposited in the open science framework repository.^81^
