# Supplementary material for: Effects of formalin fixation on polarimetric properties of brain tissue: fresh or fixed?
Source: Neurophotonics. 2023 May 24;10(2):025009. doi: 10.1117/1.NPh.10.2.025009 (PMC10207713; doi:10.1117/1.NPh.10.2.025009)
Supplement: Supplementary file 1 [file NPh_010_025009_SD001.pdf]

Effects of formalin fixation on polarimetric properties of brain tissue: fresh or fixed?

Romain Gros[1,2*], Omar Rodríguez-Núñez[3], Leonard Felger[3], Stefano Moriconi[4], Richard McKinley[4], Angelo Pierangelo[5], Tatiana Novikova[5], Erik Vassella[1], Philippe Schucht[3], Ekkehard Hewer[6], Theoni Maragkou[1]

[1] Institute of Tissue medicine and Pathology, University of Bern, 3010, Bern, Switzerland

[2] Graduate School for Cellular and Biomedical Sciences, University of Bern, 3010, Bern, Switzerland

[3] Department of Neurosurgery, Inselspital, Bern University Hospital, University of Bern, 3010, Bern, Switzerland

[4] Support Center for Advanced Neuroimaging (SCAN), University Institute of Diagnostic and Interventional Radiology, University of Bern, Inselspital, Bern University Hospital, 3010 Bern, Switzerland

[5] LPICM, CNRS, Ecole polytechnique, IP Paris, Palaiseau, 91128, France

[6] Institute of Pathology, Lausanne University Hospital and University of Lausanne, Lausanne, Switzerland

Corresponding author:

Romain Gros: romain.gros@unibe.ch

SUPPLEMENTARY MATERIAL

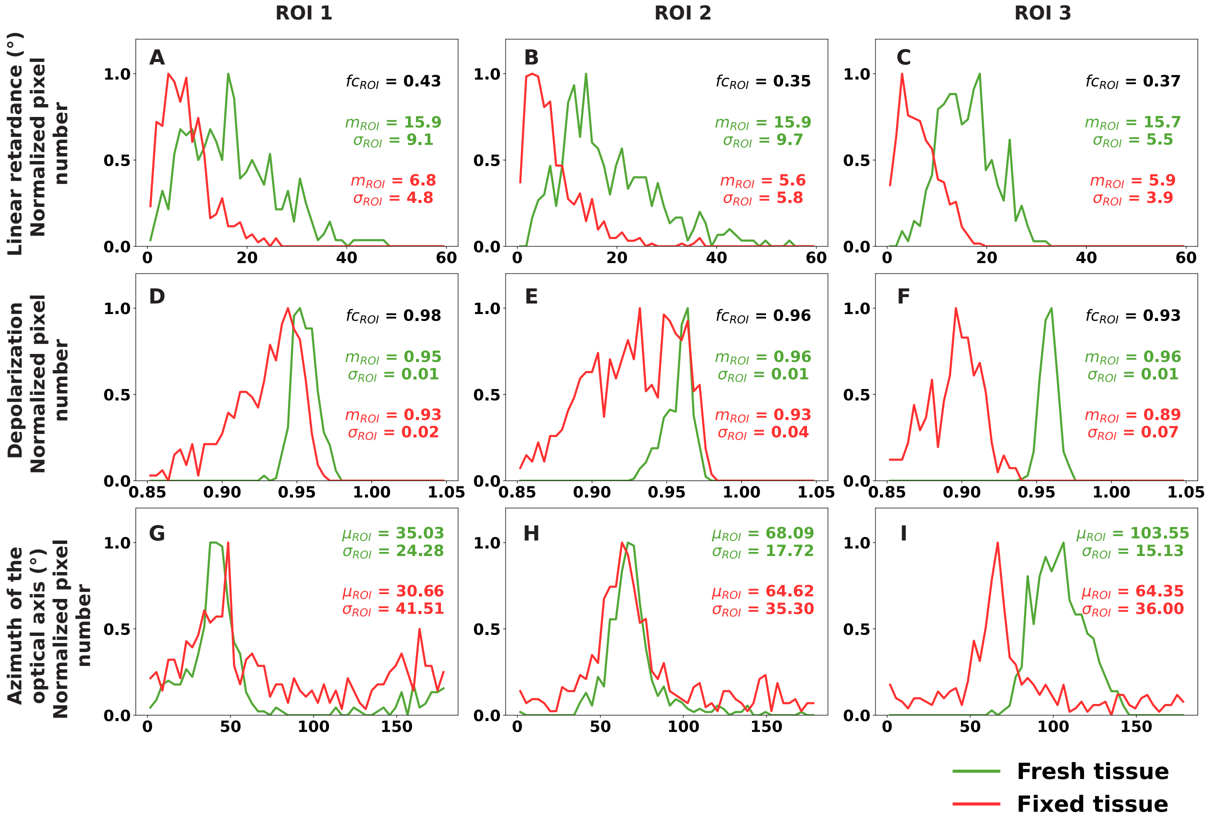

Figure S1: Distribution of the polarimetric parameters within single ROIs. Representation of the distribution of the linear retardance (A-C), depolarization (D-F) and azimuth of the optical axis (G-I) within single ROIs before and after formalin fixation. The distributions are represented for 3 different ROIs (each corresponding to one column). The distributions of the linear retardance and depolarization appear skewed, motivating the decision to use the median as statistical descriptor.

Linear retardance in grey matter					
**Time**	**Mean**	**U**	n_1	n_2	p_{val}
**0 hrs**	8.76 ± 4.69	1983	85	100	$4{\times}10^{-10}$
**+12hrs**	5.51 ± 3.67	3612	85	85	1
**+24hrs**	5.55 ± 3.39	3485	85	87	0.51
**+36hrs**	4.98 ± 2.93	3806	85	86	0.64
**+48hrs**	6.50 ± 4.74	3330	85	92	0.089
**+7days**	5.39 ± 3.31	3693	85	89	0.79

Linear retardance in white matter					
**Time**	**Mean**	**U**	n_1	n_2	p_{val}
**0 hrs**	19.79 ± 10.30	2453	94	100	$9{\times}10^{-9}$
**+12hrs**	12.10 ± 6.58	4418	94	94	1
**+24hrs**	12.32 ± 5.98	4067	94	91	0.56
**+36hrs**	11.54 ± 5.93	4441	94	92	0.75
**+48hrs**	12.28 ± 6.92	4440	94	96	0.85
**+7days**	10.62 ± 5.07	5010	94	96	0.19

Table S1: Summary of the statistics for the comparison between the means of the linear retardance median values at different time points post-fixation. All the reported **U** and p values correspond to the test of the null hypothesis, stating that the mean variable is the same as the one 12 hours post -fixation.

Depolarization in grey matter					
**Time**	**Mean**	**U**	n_1	n_2	p_{val}
**0 hrs**	0.804 ± 0.046	6473	85	100	$9{\times}10^{-10}$
**+12hrs**	0.844 ± 0.042	3612	85	85	1
**+24hrs**	0.837 ± 0.057	3842	85	87	0.66
**+36hrs**	0.827 ± 0.060	4129	85	86	0.14
**+48hrs**	0.830 ± 0.062	4385	85	92	0.16
**+7days**	0.841 ± 0.050	3721	85	89	0.85

Depolarization in white matter					
**Time**	**Mean**	**U**	n_1	n_2	p_{val}
**0 hrs**	0.931 ± 0.029	4568	94	100	0.74
**+12hrs**	0.931 ± 0.026	4418	94	94	1
**+24hrs**	0.932 ± 0.024	4065	94	89	0.80
**+36hrs**	0.929 ± 0.025	4604	94	91	0.41
**+48hrs**	0.918 ± 0.033	5446	94	96	$8{\times}10^{-3}$
**+7days**	0.922 ± 0.028	5412	94	96	0.019

Table S2: Summary of the statistics for the comparison between the means of the depolarization median values at different time points post-fixation. All the reported **U** and p values correspond to the test of the null hypothesis, stating that the mean variable is the same as the one 12 hours post -fixation.
